# Pockets as structural descriptors of EGFR kinase conformations

**DOI:** 10.1371/journal.pone.0189147

**Published:** 2017-12-11

**Authors:** Marcia Anahi Hasenahuer, German Patricio Barletta, Sebastián Fernandez-Alberti, Gustavo Parisi, María Silvina Fornasari

**Affiliations:** Departamento de Ciencia Y Tecnología, Universidad Nacional de Quilmes, Bernal, Buenos Aires, Argentina; Wake Forest University, UNITED STATES

## Abstract

Epidermal Growth Factor Receptor (EGFR), a tyrosine kinase receptor, is one of the main tumor markers in different types of cancers. The kinase native state is mainly composed of two populations of conformers: *active* and *inactive*. Several sequence variations in EGFR kinase region promote the differential enrichment of conformers with higher activity. Some structural characteristics have been proposed to differentiate kinase conformations, but these considerations could lead to ambiguous classifications. We present a structural characterisation of EGFR kinase conformers, focused on active site pocket comparisons, and the mapping of known pathological sequence variations. A structural based clustering of this pocket accurately discriminates active from inactive, well-characterised conformations. Furthermore, this main pocket contains, or is in close contact with, ≈65% of cancer-related variation positions. Although the relevance of protein dynamics to explain biological function has been extensively recognised, the usage of the ensemble of conformations in dynamic equilibrium to represent the functional state of proteins and the importance of pockets, cavities and/or tunnels was often neglected in previous studies. These functional structures and the equilibrium between them could be structurally analysed in wild type as well as in sequence variants. Our results indicate that biologically important pockets, as well as their shape and dynamics, are central to understanding protein function in wild-type, polymorphic or disease-related variations.

## Introduction

**Fig 1:**
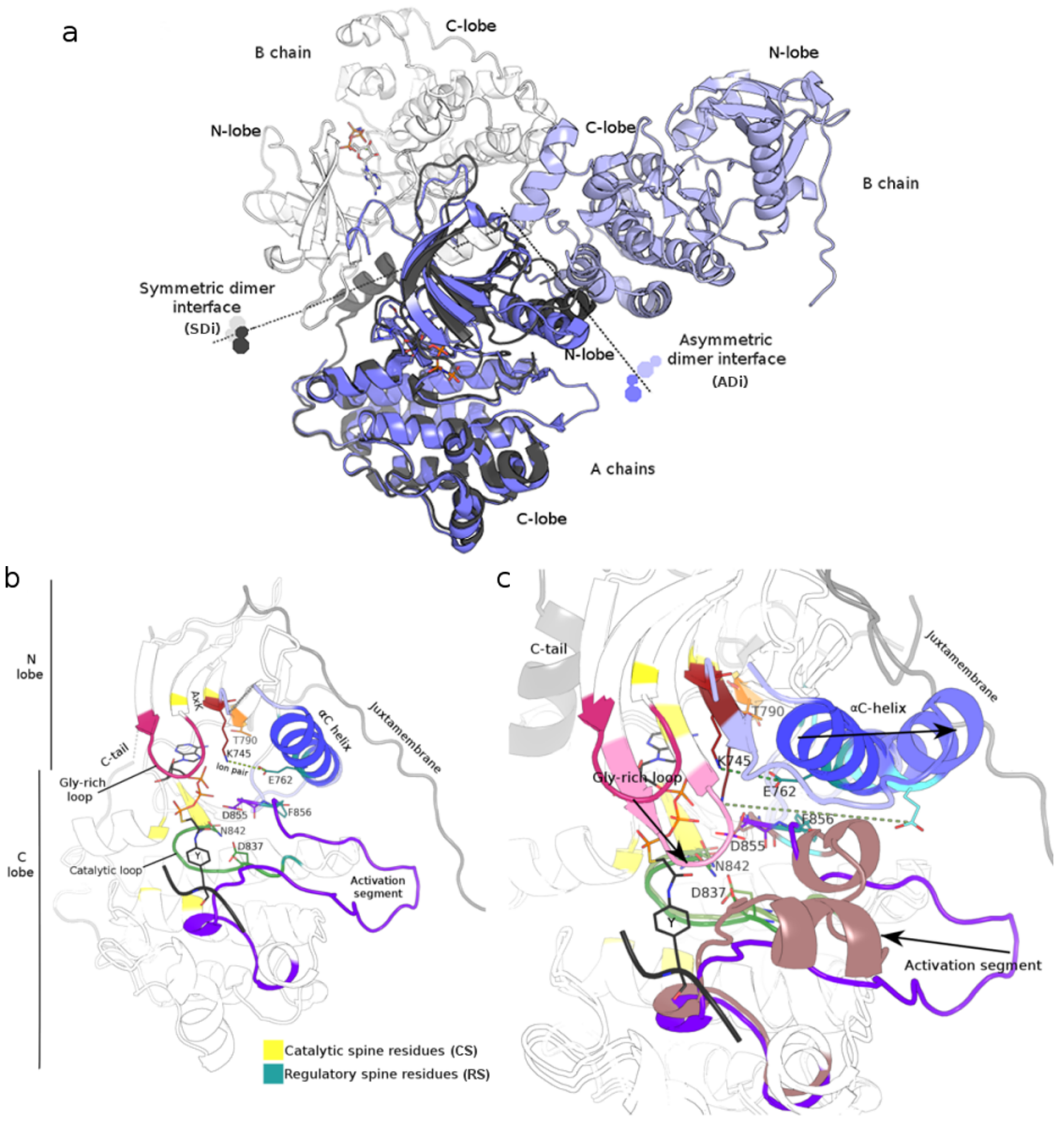
EGFR kinase dimers and key structural elements important for catalysis and regulation. (**A)** Superposition of A chains of a symmetric dimer (PDB 3GT8, in grey colours) and an asymmetric dimer (PDB 3IKA, in blue colours). Interfaces are depicted with dotted lines. (**B)** Active monomeric conformer with an ATP analog+peptide conjugate (PDB 2GS6). Key structural elements are coloured magenta (Gly-rich loop), blue (**α**C helix), violet (activation segment), green (catalytic loop), and sites in red (K745 in the AxK motif), orange (T790 gatekeeper), yellow (catalytic spine residues) and cyan (regulatory spine residues). Ion pair (salt-bridge) between K745 and E762 is depicted with a dashed green line. **(C)** Comparison of these elements in active (PDB 2GS6) and inactive (PDB 3W32) monomers following the same colour scheme, but in lighter tones. Main transitions of these elements from active to inactive are depicted with black arrows.

Conformational ensembles are nowadays increasingly used to understand protein function [1]. However, most of those studies use backbone coordinates to define open and close conformers neglecting the importance of cavities, pockets and tunnels (gates of enzymes). In the present work, pockets and cavities are considered to better characterise the alternative conformations in the kinase region of the Epidermal Growth Factor Receptor (EGFR). This tyrosine kinase receptor is one of the main tumor markers in many cancer types [2]. Its cytoplasmic region is composed by a juxtamembrane (JM), a Tyr-kinase domain, and a C-terminal intrinsically disordered tail (C-tail) target of auto-phosphorylations which triggers signals involved in different cell processes [3,4]. In different types of cancers, an increase in kinase activity and its resultant deregulation are observed [5,6]. Several single amino acid substitutions (SASs) as well as insertions and deletions located in the kinase region and detected in patients affected with different cancers, mainly non-small cell lung cancer (NSCLC), have been proposed as cause of this kinase activity enhancement [7]. In the case of the EGFR kinase domain, as in other kinases, the native state is mainly composed of two populations of conformers called *active* and *inactive* or *dormant* structures [8,9]. Moreover, it has been proposed that the stabilisation of the EGFR kinase active conformation is mediated by the formation of an asymmetric dimer (interface between the C-lobe of one subunit with the N-lobe of the other, Fig 1). Several works have reported kinase activity assays and/or their corresponding structures, allowing the generalisation of some structural and sequence features that are typical of active conformations [10–13]. Unfortunately, some of these shared structural traits are, expectedly, not easily detected in inactive conformations due to their intrinsic structural variability when compared with their active counterparts. However, in the case of kinases, some inactive conformations share common traits that have been repeatedly observed [14–16].

The identification of specific structural features of active and inactive conformations is relevant to improve our understanding of the deregulation of enzyme activity, as well as to gain knowledge on the specificity and selectivity of inhibitors [17,18]. This distinction is also important to better evaluate the impact of sequence variants. Briefly, sequence variants may cause active conformation enrichment at equilibrium due to the structural stabilisation of the active conformation [19]. Alternatively, sequence variants may also have a destabilising effect on inactive conformations, changing in both cases the ΔG barriers between conformers, with the consequent enrichment of the active form at equilibrium [20,21]. Moreover, an alteration of the inter-monomer interaction can also change enzyme activity due to equilibrium perturbation. In the case of EGFR, the study on the effects of many reported sequence variants has promoted a lot of research work in response to targeted therapy treatment decisions [22,23]. Phenotypic and clinical outcomes of several activating variants are well-known, but new ones are frequently reported as a consequence of the currently progressive extension of the sequencing of patient samples [24,25]. Thus, when it is not possible to perform an activity assay, the characterisation and eventual classification of each new sequence alteration using just sequence, structural and evolutionary information is of great interest [26]. These analyses could also, for each case, delimit the group of most appropriate inhibitors [17]. Thus, a structural description based on experimental data or derived from homology modelling, *in silico* structural analysis or docking studies may improve our understanding of the structural and/or functional effect of different reported variants [27–29].

In addition to the effect on kinase activity due to the enrichment of active conformer in the equilibrium caused by a sequence variation, small molecule kinase inhibitors show different conformer dependent mechanisms of binding. Thus, inhibitors of type I bind to active conformations, while Types I ½ and II to inactive ones. Several non-covalent inhibitors interact with the kinase ATP-binding pocket, a structure with different characteristics depending on the conformer type while others are bivalent or are allosteric [30,31], and protein allosterism also depends on the conformational ensemble of the protein [32]. The selectivity and specificity of these inhibitors also depend on the kinase sequence, structural or conformational differences being a challenge in the recognition of the specific characteristics of each particular kinase [33–35]. Briefly, and to bring the present work into focus, several distinctive characteristics of active and inactive conformations are presented, following, in all descriptions, human EGFR canonical amino acid sequence numbering (Universal Protein Resource, UniProtKB accession P00533, isoform 1, 1210 amino acids in length). Two main structural elements are usually analysed to distinguish between active and inactive kinase typical conformations. Firstly, the αC helix (positions 753–767, N-lobe) orientation: rotated inward against the N-lobe and towards the active site, this is characteristically observed in active conformers, and is crucial for kinase activity. This αC helix disposition shorts the distance between E762 and K745, allowing a stabilising ion–ion interaction (salt-bridge) between E762 of the αC helix and K745 in the β3 strand (740–747, N-lobe; a detailed description is found in Jura et al. 2011 and the references therein [10]) which interact with the α and β phosphates of ATP to anchor and orient the ATP. Secondly, in the activation segment (855–884), the Asp-Phe-Gly (DFG) motif at the beginning exhibits its aspartate in an active state conformation pointing into the ATP-binding site and coordinating a Mg^+2^ ion (only one per monomer can be observed so far, in all known crystal structures of EGFR kinase). This organisation is accompanied by an open and extended conformation of the activation loop, that is, a part of the activation segment, and is known as a DFG-in conformation. As a counterpart, several inactive kinase conformations show a DFG motif flipping towards the orientation known as DGF-out, with an almost reciprocal change in the relative orientation of D and F. In the out form, F is in the position previously occupied by aspartic acid. The change in the αC helix towards the position known as the out state has been proposed, as a general kinase activation mechanism, to be mediated by intermediate orientations, making the establishment of active or inactive αC helix orientation limits no easy task [11]. These elements are shown in Fig 1b and 1c for two representative conformations of the EGFR kinase domain (inactive PDB 3W32, active PDB 2GS6). Apart from these elements, there are others important to the stabilisation of the ATP-active site interaction, also shared by different kinases, such as the triad HRD (positions 835–837) in the catalytic loop [36]and different proposed amino acid networks [37–40]. Of these networks, two are proposed to be involved in the regulation of kinase activity: a catalytic spine (C-spine) and a regulatory spine (R-spine) [41].

Here, we examined the above described structural parameters in all human EGFR kinase domains deposited in structural databases and previously characterised in bibliography as active or inactive conformations. While several structures fulfilled all these structural criteria and, consequently, were easily classified as active or inactive, others showed both active and inactive features and, consequently, could not be unambiguously classified. Moreover, some well-characterised structures with variants proposed for a long time to constitutively stabilise the active conformation were, controversially, later reported as inactive [42]. At this point, considering the observed structural differences between conformers, in order to address some of the previously reported controversies or ambiguous conformation classifications, and to recognize the relevance of pockets, cavities and tunnels in protein function, we focused on their differential features as observed in conformational comparisons. Pockets, cavities and tunnels are structures that connect the protein surface with buried active or binding sites in proteins, and that are essential for biological activity in most proteins [43]. Their conformational changes define, for example, differential binding constants that may explain biological function, substrate specificity and important regulatory processes such as allosterism [44,45]. A slight rotation of certain given residues, usually called gatekeepers (e.g. bottleneck dynamics [46]) or larger conformational changes (e.g. conformational gating [47] and malleability [48]) are the main mechanisms controlling the transit of substrates and products to and from the protein inside. The dynamic nature of the native state, at the expense of structural differences between conformers, and their relation to changes in tunnels, pockets or cavities are essential for a complete description of protein function. Their consideration could shed light in the sometimes ambiguous conformer characterisation in proteins in general and in EGFR in particular.

In this work, a quantitative structural comparison of the pocket containing the active site (*main pocket*) allowed the correct discrimination of EGFR kinase conformations (active/inactive) taking also into account atypical conformer grouping. This comparison was performed using a hierarchical clustering based on the root mean square deviation (RMSD) of α-carbons belonging to positions of this main pocket. Even though there are several works considering pockets related to the kinase active site, quantitative structural-derived comparisons are presented here for the first time [49]. Interestingly, our findings indicate that 53 main pocket-belonging positions hold structural conformer-specific information when compared with non-pocket positions. Additionally, the mapping and characterisation of reported cancer-associated variants were also studied resulting in a notorious proportion of all the 153 kinase position–holding variants (101 [≈ 65%]) which belong or are in close contact with this main pocket. Finally, it is interesting to highlight the importance of these main pocket–shared positions in reflecting their backbone spatial constraints to regulate protein function.

## Materials and methods

### Structural conformations and sequence variants

Three-dimensional coordinates of the EGFR kinase domain conformers were retrieved from CoDNAS (http://www.codnas.com.ar/ [50]) and PDB (http://www.pdb.org [51]). Multimeric crystals were split into individual chains, resulting in a total of 103 conformers with a crystal resolution less than or equal to 3.00 Å. Available structures not already published with crystal oligomeric structures different from well-known EGFR dimeric forms or involved in hetero-oligomers were also removed.

Missing atoms of lateral chains were completed with the *complete_pdb* routine of Modeller [51,52]. Sequence variants of the EGFR kinase domain for all types of cancer were obtained from COSMIC (Catalog of Somatic Mutations in Cancer, http://cancer.sanger.ac.uk/cancergenome/projects/cosmic/ [7,53]), specifically from the targeted screen (curated) data set (version 78, September 2016).

### Pocket calculation, structural alignments and data set building

Pockets, cavities and tunnels predictions on active and inactive conformers were performed using Fpocket program [54]. Pockets related to the active site of the kinase domain, the main pocket, were manually selected by visual inspection of the active conformers PDB ids 1M14 (apo form) and 2GS6, and by considering all EGFR residues within a 5 Å radius from each atom of the ATP analog substrate–peptide conjugate in 2GS6 [55]; in this way, the main pocket of the active site was defined including 53 positions. Also, a neighborhood of close contact residues was delimited, considering residues with atoms within a 5 Å radius from each pocket amino acid. The value of 5 Å as limit to define a contact was chosen as a reasonable balance of the energetic contribution of each type of noncovalent interaction at a given distance between interacting atoms or residues [56–60].

As the rest of the pockets, cavities and tunnels were not shared by all the conformations, even within active or inactive groups, this study was centered on the main pocket. All versus all pairwise α-carbon structural alignments of kinase regions of all retrieved structures with a resolution equal or better than 3 Å and with the exclusions previously mentioned were performed with MAMMOTH [61]. To reduce redundancy and to avoid conformer over-representations, structures derived from the same work and with a global α-carbon RMSD equal or less than 0.50 Å were removed. This value is close to the estimated crystallographic method error [62]. The final set consisted of 58 structures. Also, all versus all pairwise structural alignments were performed using the main pocket positions for each structure. In addition to this, to study the biological information content of the 53 main pocket positions in the active-inactive conformer division, 1000 resamplings were done, choosing randomly 53 non-pocket positions each time. For each sample, the 53 selected positions were pairwise α-carbon structurally aligned, obtaining 1000 all vs. all RMSD matrices.

### RMSD-based clustering

In order to explore the biological information content of the main pocket, hierarchical clusterings were performed over all vs. all α-carbon RMSD values obtained taking into consideration pocket and kinase positions. Neighbor–Joining and UPGMA clustering methods taken from the Phylip package were used (Phylogeny Inference Package, version 3.7 a) [63]. Also, to study the contribution of the 53 main pocket positions to the active-inactive conformer division, 1000 α-carbon RMSD hierarchical clusterings were estimated. They were then used to find the Majority Rule Extended (MRE) [63] and Majority Rule (MR) [64] consensus clustering (data not shown) using also the Phylip package.

### Sequence variants were extracted from COSMIC and CLINVAR databases

A total of 17234 samples containing EGFR kinase sequence variants were extracted from COSMIC [65]. A comparison with Clinvar [66] information reported no differences. Of those, 16117 corresponded to NSCLC samples. Sequence variants taken from COSMIC, included 153 positions involved in SASs (missense substitutions), 47 different kinds of deletions and 25 different kinds of insertions in the kinase region. A detailed description of these sequence variants is included in S2 Table. Sequence variants in the Juxtamembrane Segment and C-tail are also included.

### Mutations mapping, DFG orientation, salt-bridge distances

The visualisation of conformers and pockets, mutation mapping and DFG orientation analysis were performed using PyMOL in the active, inactive, monomeric and dimeric conformers of the kinase domain. K745–E762 distance measurements of the N–O ion pair were performed with *ad hoc* scripts. Cut-off distances for the salt-bridge range were taken from the works of J. Thornton and R. Nussinov [67–69].

## Results

### Functional structures in EGFR

As previously mentioned, active conformations have their own structural particularities and several common features that can also be extracted from inactive conformations (Fig 1). The pocket limited by the two lobes houses the active site and is central in our comparisons. It is evident at a glance, as well as from structural alignments, that some active and inactive conformations are different; however, not all conformations are easily distinguished as active or inactive. Specific examples of conformations that exhibit several structural traits of classical active conformations and others that show inactive conformations are described in the next section. Moreover, several specific characteristics appear in some groups. The established structural parameters used to discriminate active from inactive kinase conformations were evaluated in all available EGFR kinase structures, and are: DFG orientation, the distance between the N atom of the epsilon amine group and K745 and the distance between the two oxygen atoms of the gamma carboxyl group and E762. This analysis defines more than two conformer groups, allowing different classification schemes. S1 Table includes the complete set of all available experimental EGFR kinase structures, together with the current structural parameters used to describe alternative conformations. Moreover, alternative orientations of DFG motive lateral chains and distance range between K745–E762 were defined: three orientations for D and 6 for F lateral chains together with three intervals for ion–ion distances. S1 Fig shows these alternatives graphically. As seen in this figure, structural differences can be obtained by a structural comparison of the backbone. The appearance of more than two conformer groups thus does not allow a reliable differentiation of only one active and one inactive group. This impossibility motivated us to search for an alternative structural criterion for comparisons aimed at discriminating active from inactive conformations. Pocket comparisons were consequently performed.

### EGFR main pocket definition, alignment and clustering

**Fig 2:**
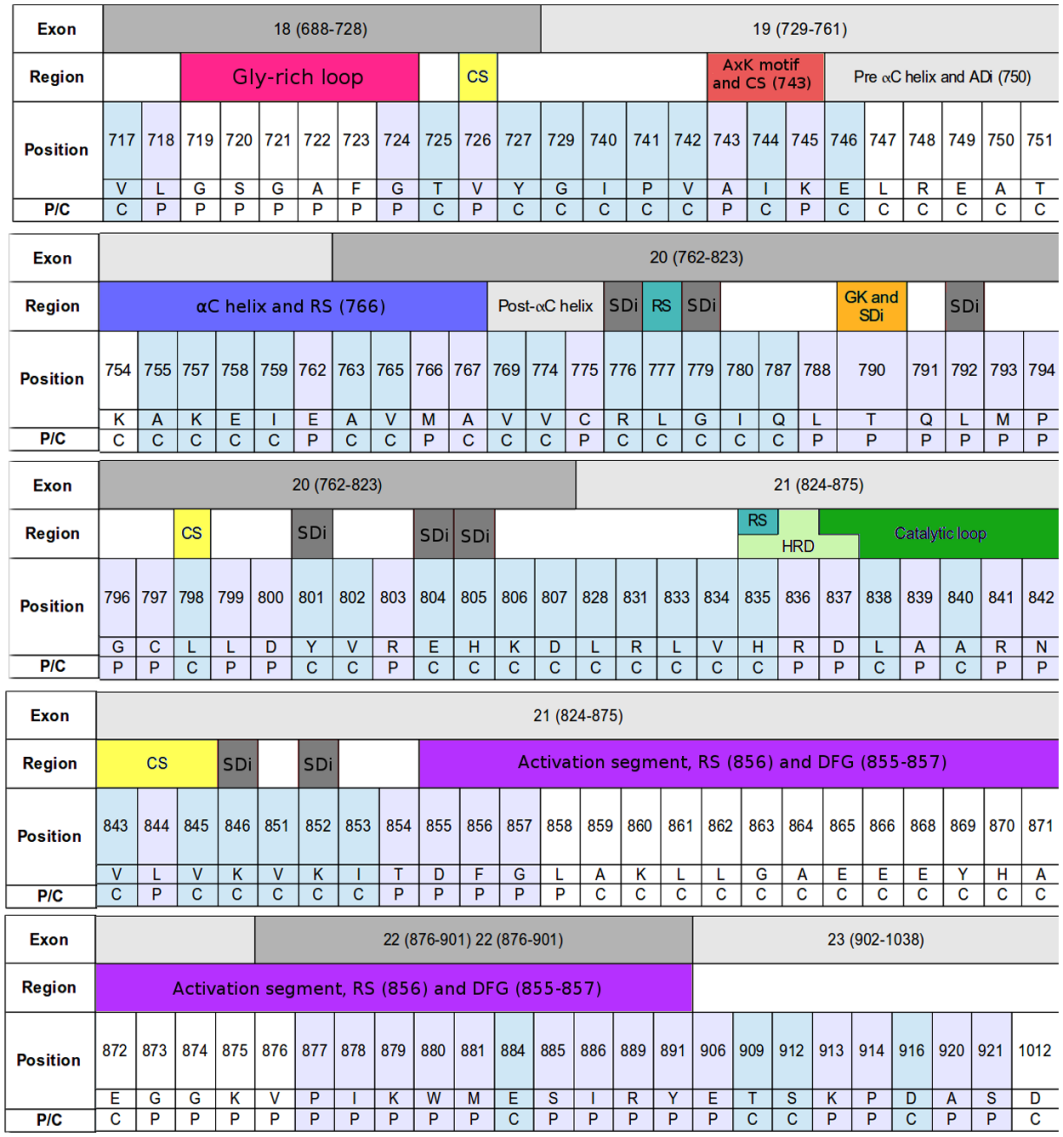
Positions belonging to and in close contact with the main pocket. Exon: shows the limits of each exon. Region: informs the conserved regions involved in catalytic control. Abbreviations: CS: catalytic spine; ADi, SDi: assymetric/symmetric dimer interface; RS: regulatory spine; GK: gatekeeper. Position, list number and residue for each position in the main pocket and close contact. P/C: depicts whether a position is considered as part of the main pocket (P) or in close contact (C). Position and P/C rows are coloured, according to the considerations in P/C, for those sites with defined coordinates in all the structures used in the present work. Those in white correspond to positions that are found as missing in at least one of these structures.

**Fig 3:**
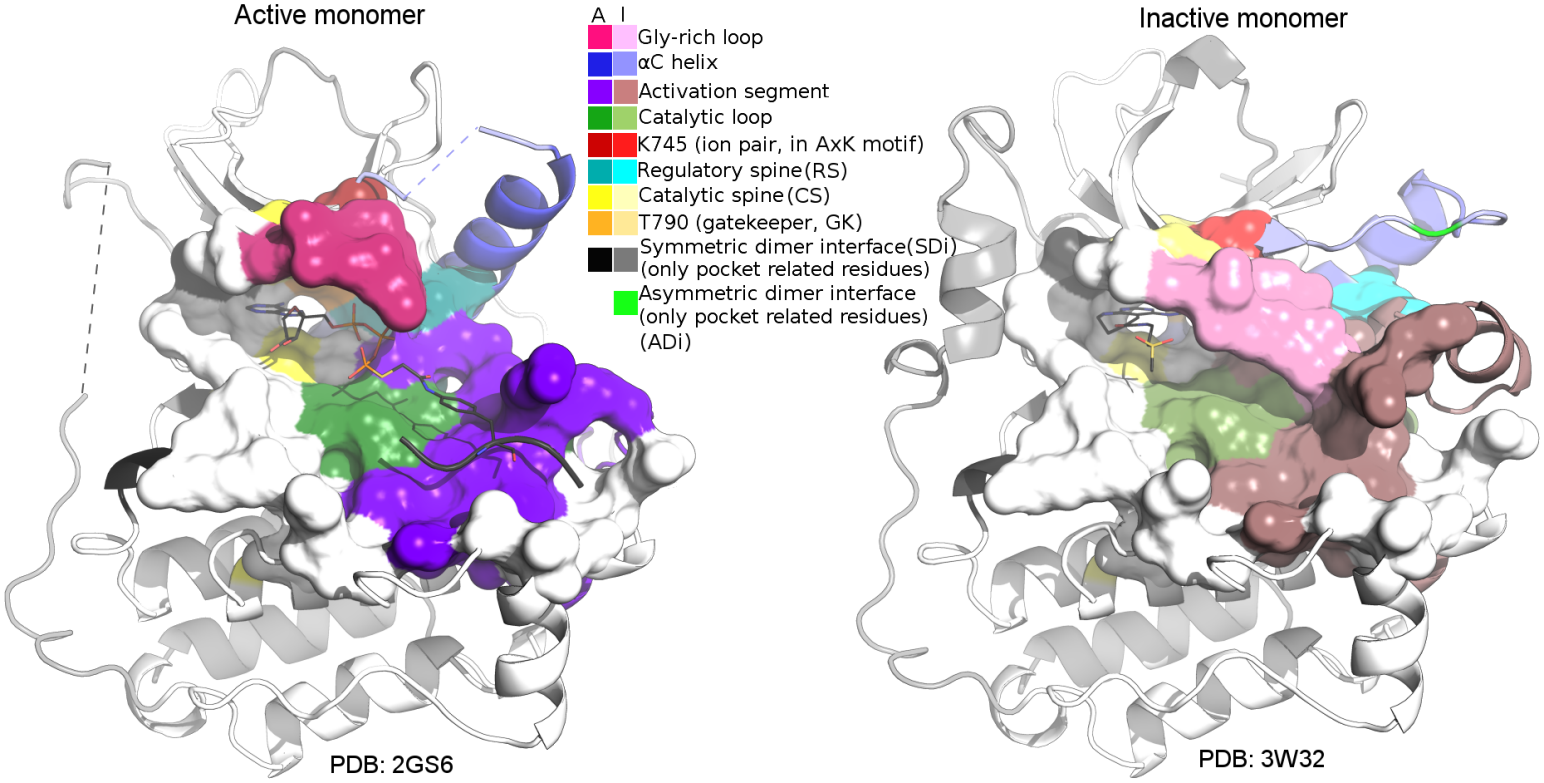
Main pocket comparison. Main pocket comparison between active (left, PDB 2GS6 with ATP analog–peptide conjugate) and inactive (right, PDB 3W32 with pyrimido [4,5-b]azepine-derived inhibitor) monomers, following the same colouring scheme as in Figs 1 and 2 (active: A colours, inactive: I colours). The 53 main pocket positions are represented as surfaces in both monomers. For the symmetric and asymmetric interface residues, only those included or in close contact to the main pocket are depicted in black and bright green, respectively.

The main pocket, which involves 53 positions, was defined using structural and biological information, as described in Materials and Methods. Unlike other pockets, tunnels or cavities detected in the kinase region of the EGFR, this main pocket is present and clearly distinguishable in all conformations. Because of that, the other pockets as well as different cavities and sporadic or short tunnels were not considered. Additionally, positions involved in noncovalent interactions with main pocket positions (contact positions) were registered (67). Main pocket and contact positions are included in Fig 2, together with their corresponding exon number and the distinctive structural aspects of the region where they belong. Fig 3 includes a main pocket structural comparison. Regarding main-pocket positions, an important proportion of them have defined coordinates in all of the structures. Main pocket positions missing in at least one conformer are: S719-F723 in Gly-rich loop, L858 and G873-V876 in the activation segment. Both are flexible regions of the kinase domain; Gly-rich forms a cover on top of the ATP and bridges to its γ-phosphate positioning it for the phosphoryl transfer.

**Fig 4:**
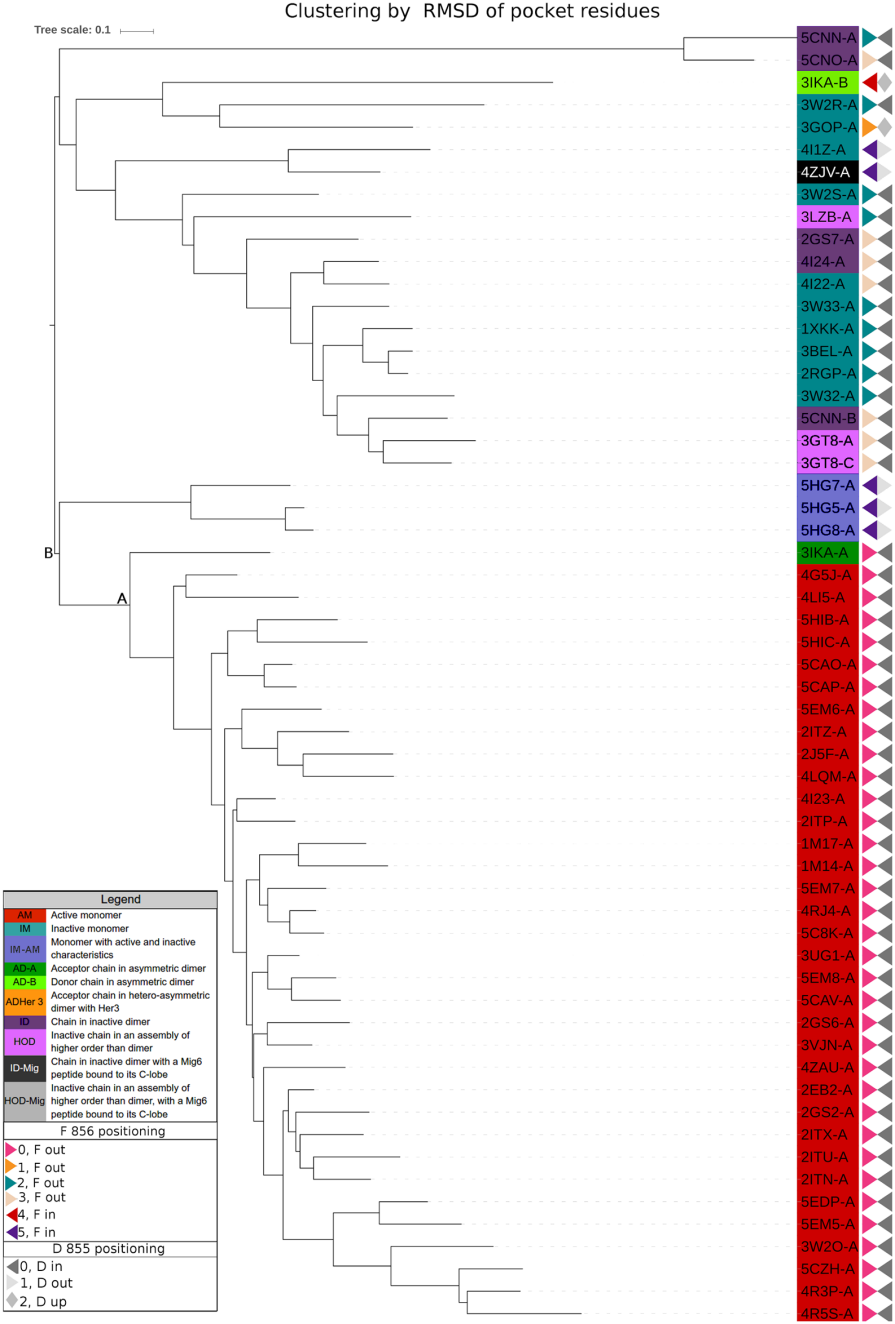
Hierarchical clustering. Hierarchical clustering based on the α-carbon RMSD, using only main pocket residues. Node A divides active and inactive conformations. Node B includes a particular group (PDBids: 5HG5:A; 5HG7:A; 5HG8:A) as part of active conformations’ group (see *EGFR main pocket definition*, *alignment and clustering* section in Results).

The final data set with a resolution equal or better than 3 Å included 58 structures (selection explained in the Materials and methods section). Fig 4 includes the Neighbor–Joining (N–J)-based hierarchical clustering of α-carbon RMSD derived from the pairwise structural alignment of the positions belonging to the main pocket and, similarly S2 Fig shows the hierarchical clustering of α-carbon RMSD of all kinase positions. The Unweighted Pair Group Method with Arithmetic Mean (UPGMA)-based clusterings are very similar to N–J and are not shown. As already mentioned, the previous classification of conformations as active or inactive was taken from bibliography. The different colours reflect different groups of conformers, as defined in S1 Table, according to their structural particularities.

It is interesting to note that node A using pocket-based clustering mainly divides active from inactive EGFR conformations. Alternatively, node B divides conformations leaving structures 5HG7, 5HG5 and 5HG8 grouped with the active ones. In order to explore the biological information content of the main pocket positions, as explained in Materials and Methods, we performed 1000 resamplings selecting at random 53 non-pocket positions that were later structurally aligned and clustered. The1000 clusters that were obtained were used to find the Majority Rule Extended (MRE) and Majority Rule (MR) consensus clustering (data not shown). Node A shows a statistical support of 0.39 and node B of 0.21, even lower than node A support. Moreover, these nodes are absent in Majority Rule (MR) Consensus clustering (data not shown). These results highlight the biological information contained in main pocket positions in reference to their capacity to differentiate active from inactive conformations.

As it was previously mentioned, different groups of conformers have particular characteristics, sharing structural features both with classical active or inactive conformations or having their own particularities. For example, the group represented by the structures reported by Cheng et al. [17], PDB ids 5HG7, 5HG5, 5HG8 and 5HG9, exhibit a classic DFG-out conformation in the presence of ligands; however, their activation loop is in a state nearly identical to the one in the active form. In pocket clustering groups, these conformers are separated from the rest of the inactive group and also from the rest of the active conformations. Nevertheless, in complete sequence clustering, they appear close to and share a node with three (uncommon) inactive conformations, 3IKA_B, 3GOP_A and 3W2R_A, but do not share the orientation of the lateral chains of D845 and F846. 3IKA_B is the *activator* or *donor* chain in the asymmetric dimer and it packs its C-lobe against the N-lobe of 3IKA_A. As it was already mentioned, it has been proposed that the stabilisation of the EGFR kinase active conformation is mediated by the formation of this asymmetric dimer [16]. However, unfortunately, 3IKA is the only asymmetric dimer representant that we could include in RMSD clustering; because of lower resolutions, 2JIT, 2JIU, 4G5P and 4LL0 were discarded. This *donor* chains, also show clear differences from all the other conformers when superposing their backbones (data not shown). Unfortunately, even if these PDBs are the only ones showing this complete configuration as asymmetric dimers in an asymmetric unit of a crystal, they all harbor the T790M variation. To explore the influence of these conformations in the native state in wild type as well as in T790M and other possible variants, good resolution asymmetric wild type dimer crystals are needed.

### A significant number of disease-related EGFR kinase SASs belong to the main pocket

A total of 17234 samples containing EGFR kinase sequence variants were extracted from COSMIC [65]; a comparison with Clinvar data [66] did not report changes. From these, 16117 correspond to NSCLC samples. In terms of the different variations that are represented, sequence variants taken from COSMIC include, in the kinase region, 153 positions involved in SASs (single amino acid or missense substitutions), 47 different kinds of deletions and 25 different kind of insertions. A detailed description of these sequence variants is included in S2 Table, together with sequence variants located in the JM segment and C-tail. A notorious proportion of all 15 kinase position-holding variants, 101 (≈65%), belong or are in close contact with the main pocket. Thirty-six of the 101 correspond to main pocket positions for a total of 53 main pocket positions (≈68% main pocket positions are involved in disease-associated sequence variants) and 65 from 67 main pocket contact positions are also affected by variants (97%). The calculation of this proportion for the remainder of the kinase positions (excluding the main pocket and its contact positions) gives a percentage of ≈44% (68 positions affected by disease-associated variants over 153 kinase positions), showing a significant enrichment of the main pocket and its contacts in positions affected by disease-related sequence variants. This enrichment reflects the functional importance of both the main pocket and its contact positions, affecting protein activity both under normal physiological conditions as well as during disease [70,71].

## Discussion

The well-recognized importance of protein dynamics, the existence of an ensemble of conformations in the native state of a protein [1], and changes in pockets’ structural features [43] to improve our understanding of protein function make them essential aspects to take into account in normal as well as in disease-related states. In terms of health care, for some well-characterised pathologies at the molecular level, patient exon sequencing is, nowadays, conducted much more frequently than in the past and provides very valuable, but not always conclusive, information. Increasing our knowledge on protein function underlying mechanisms would certainly have an impact on our understanding of sequence variants effects on patients.

**Fig 5:**
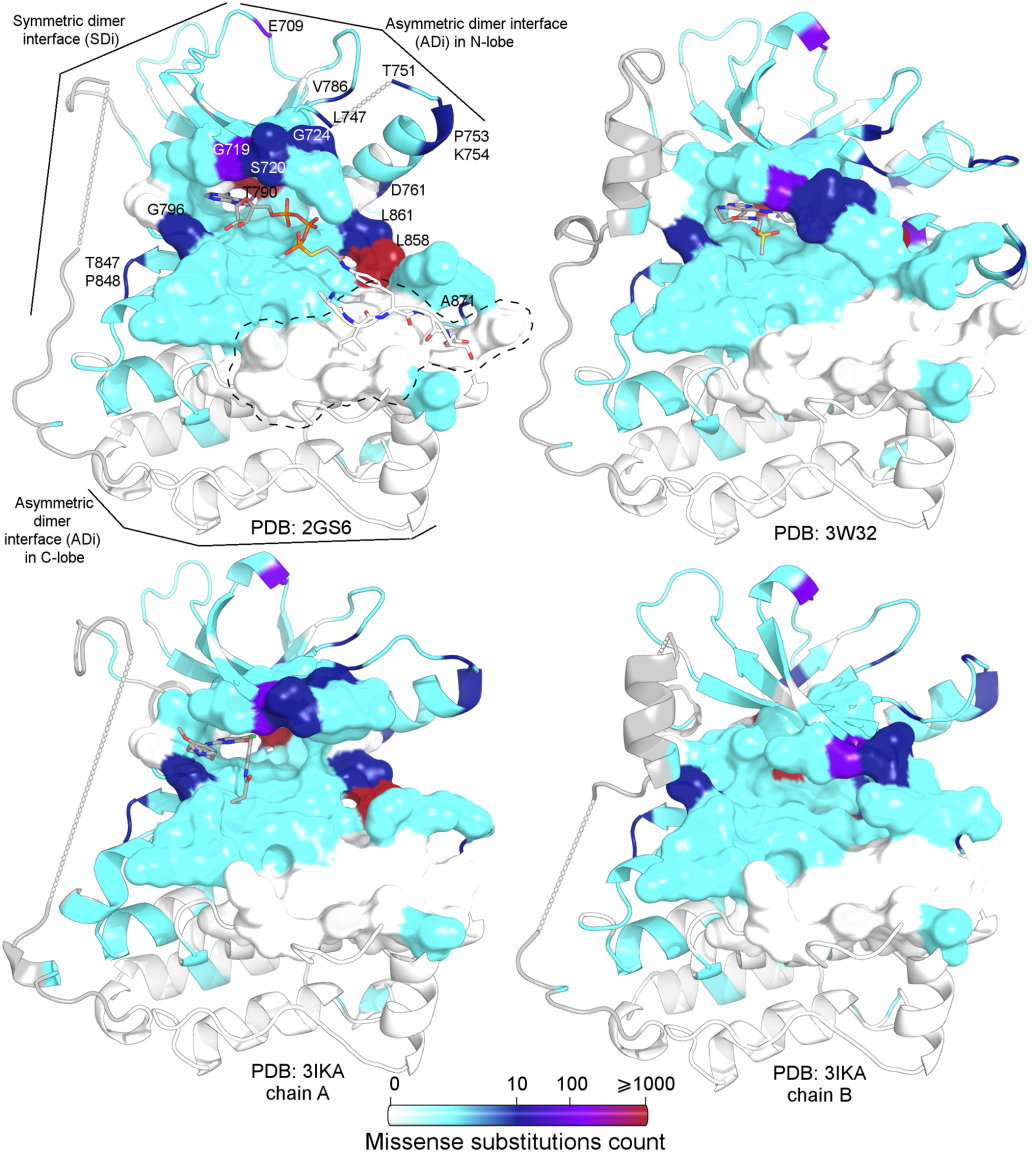
Variant site mapping over kinase domain conformers. The main pocket is represented as a surface. From left to right and from top to bottom: active monomer with ATP analog–peptide conjugate (PDB id 2GS6), inactive monomer with pyrimido[4,5-b]azepine-derived inhibitor (PDB id 3W32), active chain in asymmetric dimer with WZ4002 irreversible inhibitor (PDB id 3IKA_A), and inactive chain in asymmetric dimer (PDB id 3IKA_B). The most frequently affected sites (those in red, violet and dark blue) map in the catalytic pocket and are found in particular regions: the activation segment (with the classical L858R), the Gly-rich loop, and the region that connects both lobes of the kinase. Interestingly, most of the residues that serve as the docking site for the substrate peptide are not affected by variations (circled with a dashed black line). The total number of missense substitutions observed in COSMIC for each position is listed in S2 Table, column *Gen_var_count_ocurrence*. Missing regions are connected with straight dashed lines.

Although there are well-characterised sequence variants in terms of drug response in EGFR, serious limitations in treatment decisions appear in some cases as a result of the incomplete characterisation of those previously unreported or with controversial classification. In the present work, we studied the structures of previous experimentally determined EGFR kinase domain structures, including the analysis of pockets and cavities. Moreover, all the cancer related sequence variants included in well-curated databases were structurally mapped in different EGFR kinase domain conformations. We found that it is possible to discriminate previously reported conformations as active or inactive, as well as subsets with structural particularities, by performing a main pocket structural comparison using α-carbon RMSD-based hierarchical clusterings. Additionally, ≈65% of kinase positions with reported variants in patients affected by cancer are in, or in close contact to, this main pocket. The enrichment in disease-related variants of the main pocket position and its contacts compared to the rest of the kinases reflects their functional importance and their putative effect on protein activity in disease. Fig 5 includes a map of cancer-related variations in the EGFR kinase, reflecting how these are enriched in the main pocket.

Previously established conformation classification criteria were sometimes not conclusive due to the presence of intermediate positions, distances, orientations or angles [72]. It is also interesting to note that hierarchical clusterings, taking into account all kinase or main pocket positions, are similar but with some differences. However, the most significant finding of the present work is that only 53 main pocket positions contain the structural information necessary to discriminate active from inactive conformations, also allowing the grouping of atypical conformations. This finding reflects both the biological meaning and the importance of pockets in protein function and their relationship with reported disease-related sequence variants. Thus, only 53 specific positions entail functional significant information, sustained by the low support of nodes A and B as a result of the resampling analysis of 1000 replicates performed after taking 53 positions at random over a total of ≈220 kinase non-pocket positions. Moreover, of these 53 main pocket positions, an important fraction, 44 (≈ 83%), are structurally defined in all conformers. This observation reflects, together with the fact that the main pocket was the only recognizable one in all the conformers, the structural importance of these positions and their structural constraints. The information content rests, in addition, in backbone coordinates.

It is noticeable that our results agree with the conclusions of previous works. Firstly, conformations with a structural organisation that is intermediate between classically active or inactive exist [17,73]. Secondly, several conformations with sequence variants, L858R and/or T790M, belong to the inactive group according to the work of Gajiwala et al. [74]. In their work, thermodynamic stability analysis of these structures supported their conclusions. These sequence variants could be activating because of conformational equilibrium displacement not being necessary to assume that the kinase adopts an active conformation (constitutively) to explain their impact on kinase activity. A ligand’s presence, its concentration and the environmental physicochemical conditions should also alter conformer populations, displacing the equilibrium of alternative conformations of L858R and/or T790M variants which, by themselves, would not be able to significantly shift the conformational equilibrium towards active forms.

Even though several groups have studied pockets related to the kinase active site, this work presents, for the first time, quantitative calculations using main pocket structural comparisons to allow a better discrimination of conformations. This work extends the quantitative study of conformers with different activities. Moreover, this analysis may help to better structurally characterise and, consequently, distinguish different sequence variants which could impact on decisions related to patient treatment and the design and selection of inhibitors for disease.

## Supporting information

S1 Fig Alternative orientations of lateral chains of DFG residues. Backbones are coloured according to the groups defined by F856 orientations (see S1 Table, column “dFg”). **(A)** D855 in—D855 out. (Dfg). Superposition of backbones of DFG motif and catalytic loop. Mg2+ ions correspond to the ones observed in inactive chains from symmetric dimers (PDB ids 3GT8 and 2GS7, as orange and yellow balls respectively) and active chains in asymmetric hetero-dimers (PDB ids 4RIX, 4RIY, 4RIW, as green balls). **(B)** Different D855 orientations (Dfg). IN, pointing to Mg2+ in active site. OUT and UP, two different orientations pointing away from the active site. **(C)** Alternative F856 positions (dFg). Numbers 0 to 5 represent the different categories used to divide conformations (a more detailed description is included in S1 Table). **(D)** G857 positions (dfG).
(TIF)

S2 Fig Hierarchical clustering based on the RMSD, including complete kinase residues. (TIF)

S1 Table Complete set of all available experimental EGFR kinase structures and structural characterization. (XLS)

S2 Table COSMIC reported variants and structural related information. **(A)** Non-synonymous missense substitutions. **(B)** Deletions. **(C)** Insertions.
(XLS)

## References

[1] Wei G, Xi W, Nussinov R, Ma B. Protein Ensembles: How Does Nature Harness Thermodynamic Fluctuations for Life? The Diverse Functional Roles of Conformational Ensembles in the Cell. Chem Rev. 2016; acs.chemrev.5b00562. 10.1021/acs.chemrev.5b00562 PMC6407618 26807783

[2] Henry NL, Lynn Henry N, Hayes DF. Cancer biomarkers. Mol Oncol. 2012;6: 140–146. doi: 10.1016/j.molonc.2012.01.010 22356776 10.1016/j.molonc.2012.01.010 PMC5528374

[3] Schlessinger J. Cell Signaling by Receptor Tyrosine Kinases. Cell. 2000;103: 211–225. 11057895 10.1016/s0092-8674(00)00114-8

[4] Lemmon MA, Schlessinger J. Cell Signaling by Receptor Tyrosine Kinases. Cell. 2010;141: 1117–1134. doi: 10.1016/j.cell.2010.06.011 20602996 10.1016/j.cell.2010.06.011 PMC2914105

[5] Arteaga CL, Engelman JA. ERBB receptors: from oncogene discovery to basic science to mechanism-based cancer therapeutics. Cancer Cell. 2014;25: 282–303. doi: 10.1016/j.ccr.2014.02.025 24651011 10.1016/j.ccr.2014.02.025 PMC4018830

[6] Kalia M, Madhu K. Biomarkers for personalized oncology: recent advances and future challenges. Metabolism. 2015;64: S16–S21. doi: 10.1016/j.metabol.2014.10.027 25468140 10.1016/j.metabol.2014.10.027

[7] Forbes SA, Beare D, Gunasekaran P, Leung K, Bindal N, Boutselakis H, et al COSMIC: exploring the world’s knowledge of somatic mutations in human cancer. Nucleic Acids Res. 2015;43: D805–11. doi: 10.1093/nar/gku1075 25355519 10.1093/nar/gku1075 PMC4383913

[8] Ferguson KM. Active and inactive conformations of the epidermal growth factor receptor. Biochem Soc Trans. 2004;32: 742–745. doi: 10.1042/BST0320742 15494003 10.1042/BST0320742

[9] Kornev AP, Taylor SS. Defining the conserved internal architecture of a protein kinase. Biochim Biophys Acta. 2010;1804: 440–444. doi: 10.1016/j.bbapap.2009.10.017 19879387 10.1016/j.bbapap.2009.10.017 PMC3435107

[10] Jura N, Zhang X, Endres NF, Seeliger MA, Schindler T, Kuriyan J. Catalytic control in the EGF receptor and its connection to general kinase regulatory mechanisms. Mol Cell. 2011;42: 9–22. doi: 10.1016/j.molcel.2011.03.004 21474065 10.1016/j.molcel.2011.03.004 PMC3175429

[11] Kornev AP, Haste NM, Taylor SS, Eyck LFT. Surface comparison of active and inactive protein kinases identifies a conserved activation mechanism. Proc Natl Acad Sci U S A. 2006;103: 17783–17788. doi: 10.1073/pnas.0607656103 17095602 10.1073/pnas.0607656103 PMC1693824

[12] Valley CC, Arndt-Jovin DJ, Karedla N, Steinkamp MP, Chizhik AI, Hlavacek WS, et al Enhanced dimerization drives ligand-independent activity of mutant epidermal growth factor receptor in lung cancer. Mol Biol Cell. 2015;26: 4087–4099. doi: 10.1091/mbc.E15-05-0269 26337388 10.1091/mbc.E15-05-0269 PMC4710239

[13] Kancha RK, Peschel C, Duyster J. The epidermal growth factor receptor-L861Q mutation increases kinase activity without leading to enhanced sensitivity toward epidermal growth factor receptor kinase inhibitors. J Thorac Oncol. 2011;6: 387–392. doi: 10.1097/JTO.0b013e3182021f3e 21252719 10.1097/JTO.0b013e3182021f3e

[14] Shan Y, Arkhipov A, Kim ET, Pan AC, Shaw DE. Transitions to catalytically inactive conformations in EGFR kinase. Proc Natl Acad Sci U S A. 2013;110: 7270–7275. doi: 10.1073/pnas.1220843110 23576739 10.1073/pnas.1220843110 PMC3645566

[15] Huse M, Morgan H, John K. The Conformational Plasticity of Protein Kinases. Cell. 2002;109: 275–282. 12015977 10.1016/s0092-8674(02)00741-9

[16] Zhang X, Xuewu Z, Jodi G, Kui S, Cole PA, John K. An Allosteric Mechanism for Activation of the Kinase Domain of Epidermal Growth Factor Receptor. Cell. 2006;125: 1137–1149. doi: 10.1016/j.cell.2006.05.013 16777603 10.1016/j.cell.2006.05.013

[17] Cheng H, Hengmiao C, Nair SK, Murray BW, Chau A, Simon B, et al Discovery of 1-(3R,4R)-3-[(5-Chloro-2-[(1-methyl-1H-pyrazol-4-yl)amino]-7H-pyrrolo[2,3-d]pyrimidin-4-yloxy)methyl]-4-methoxypyrrolidin-1-ylprop-2-en-1-one (PF-06459988), a Potent, WT Sparing, Irreversible Inhibitor of T790M-Containing EGFR Mutants. J Med Chem. 2016;59: 2005–2024. doi: 10.1021/acs.jmedchem.5b01633 26756222 10.1021/acs.jmedchem.5b01633

[18] Kumar A, Petri ET, Halmos B, Boggon TJ. Structure and Clinical Relevance of the Epidermal Growth Factor Receptor in Human Cancer. J Clin Oncol. 2008;26: 1742–1751. doi: 10.1200/JCO.2007.12.1178 18375904 10.1200/JCO.2007.12.1178 PMC3799959

[19] Yun C-H, Boggon TJ, Li Y, Woo MS, Greulich H, Meyerson M, et al Structures of lung cancer-derived EGFR mutants and inhibitor complexes: mechanism of activation and insights into differential inhibitor sensitivity. Cancer Cell. 2007;11: 217–227. doi: 10.1016/j.ccr.2006.12.017 17349580 10.1016/j.ccr.2006.12.017 PMC1939942

[20] Kumar S, Ma B, Tsai CJ, Sinha N, Nussinov R. Folding and binding cascades: dynamic landscapes and population shifts. Protein Sci. 2000;9: 10–19. doi: 10.1110/ps.9.1.10 10739242 10.1110/ps.9.1.10 PMC2144430

[21] James LC, Tawfik DS. Conformational diversity and protein evolution–a 60-year-old hypothesis revisited. Trends Biochem Sci. 2003;28: 361–368. doi: 10.1016/S0968-0004(03)00135-X 12878003 10.1016/S0968-0004(03)00135-X

[22] Zhang Z, Zhenfeng Z, Stiegler AL, Boggon TJ, Susumu K, Balazs H. EGFR-mutated lung cancer: a paradigm of molecular oncology. Oncotarget. 2010;1: 497–514. doi: 10.18632/oncotarget.186 21165163 10.18632/oncotarget.186 PMC3001953

[23] Russo A, Franchina T, Ricciardi GRR, Picone A, Ferraro G, Zanghì M, et al A decade of EGFR inhibition in EGFR-mutated non small cell lung cancer (NSCLC): Old successes and future perspectives. Oncotarget. 2015;6: 26814–26825. doi: 10.18632/oncotarget.4254 26308162 10.18632/oncotarget.4254 PMC4694955

[24] Forbes SA, Dave B, Prasad G, Kenric L, Charambulos B, Minjie D, et al Abstract 62: COSMIC: Combining the world’s knowledge of somatic mutation in human cancer. Cancer Res. 2015;75: 62–62.25398440

[25] Lindeman NI, Cagle PT, Beasley MB, Chitale DA, Dacic S, Giaccone G, et al Molecular testing guideline for selection of lung cancer patients for EGFR and ALK tyrosine kinase inhibitors: guideline from the College of American Pathologists, International Association for the Study of Lung Cancer, and Association for Molecular Pathology. Arch Pathol Lab Med. 2013;137: 828–860. doi: 10.5858/arpa.2012-0720-OA 23551194 10.5858/arpa.2012-0720-OA PMC4162344

[26] Marks DS, Hopf TA, Sander C. Protein structure prediction from sequence variation. Nat Biotechnol. 2012;30: 1072–1080. doi: 10.1038/nbt.2419 23138306 10.1038/nbt.2419 PMC4319528

[27] Tramontano A, Anna T. The role of molecular modelling in biomedical research. FEBS Lett. 2006;580: 2928–2934. doi: 10.1016/j.febslet.2006.04.011 16647064 10.1016/j.febslet.2006.04.011

[28] Dixit A, Verkhivker GM. Structure-functional prediction and analysis of cancer mutation effects in protein kinases. Comput Math Methods Med. 2014;2014: 653487 doi: 10.1155/2014/653487 24817905 10.1155/2014/653487 PMC4000980

[29] Hasenahuer MA, Gustavo P, Marien G, Alberto L, Bramuglia GF, Fornasari MS. Twenty-One Novel EGFR Kinase Domain variants in Patients with Nonsmall Cell Lung Cancer. Ann Hum Genet. 2015;79: 385–393. doi: 10.1111/ahg.12127 26420346 10.1111/ahg.12127

[30] Roskoski R Jr. Classification of small molecule protein kinase inhibitors based upon the structures of their drug-enzyme complexes. Pharmacol Res. 2016;103: 26–48. doi: 10.1016/j.phrs.2015.10.021 26529477 10.1016/j.phrs.2015.10.021

[31] Wang Q, Zorn JA, Kuriyan J. A structural atlas of kinases inhibited by clinically approved drugs. Methods Enzymol. 2014;548: 23–67. doi: 10.1016/B978-0-12-397918-6.00002-1 25399641 10.1016/B978-0-12-397918-6.00002-1

[32] Tsai C-J, Nussinov R. A unified view of “how allostery works.” PLoS Comput Biol. 2014;10: e1003394 doi: 10.1371/journal.pcbi.1003394 24516370 10.1371/journal.pcbi.1003394 PMC3916236

[33] Müller S, Chaikuad A, Gray NS, Knapp S. The ins and outs of selective kinase inhibitor development. Nat Chem Biol. 2015;11: 818–821. doi: 10.1038/nchembio.1938 26485069 10.1038/nchembio.1938

[34] Fabbro D. 25 years of small molecular weight kinase inhibitors: potentials and limitations. Mol Pharmacol. 2015;87: 766–775. doi: 10.1124/mol.114.095489 25549667 10.1124/mol.114.095489

[35] Vijayan RSK, He P, Modi V, Duong-Ly KC, Ma H, Peterson JR, et al Conformational analysis of the DFG-out kinase motif and biochemical profiling of structurally validated type II inhibitors. J Med Chem. 2015;58: 466–479. doi: 10.1021/jm501603h 25478866 10.1021/jm501603h PMC4326797

[36] Knighton DR, Zheng JH, Ten Eyck LF, Ashford VA, Xuong NH, Taylor SS, et al Crystal structure of the catalytic subunit of cyclic adenosine monophosphate-dependent protein kinase. Science. 1991;253: 407–414. 1862342 10.1126/science.1862342

[37] Hemmer W, McGlone M, Tsigelny I, Taylor SS. Role of the glycine triad in the ATP-binding site of cAMP-dependent protein kinase. J Biol Chem. 1997;272: 16946–16954. 9202006 10.1074/jbc.272.27.16946

[38] Taylor SS, Kornev AP. Protein kinases: evolution of dynamic regulatory proteins. Trends Biochem Sci. 2011;36: 65–77. doi: 10.1016/j.tibs.2010.09.006 20971646 10.1016/j.tibs.2010.09.006 PMC3084033

[39] James KA, Verkhivker GM. Structure-based network analysis of activation mechanisms in the ErbB family of receptor tyrosine kinases: the regulatory spine residues are global mediators of structural stability and allosteric interactions. PLoS One. 2014;9: e113488 doi: 10.1371/journal.pone.0113488 25427151 10.1371/journal.pone.0113488 PMC4245119

[40] Hu J, Ahuja LG, Meharena HS, Kannan N, Kornev AP, Taylor SS, et al Kinase regulation by hydrophobic spine assembly in cancer. Mol Cell Biol. 2015;35: 264–276. doi: 10.1128/MCB.00943-14 25348715 10.1128/MCB.00943-14 PMC4295384

[41] Ten Eyck LF, Taylor SS, Kornev AP. Conserved spatial patterns across the protein kinase family. Biochim Biophys Acta. 2008;1784: 238–243. doi: 10.1016/j.bbapap.2007.11.002 18067871 10.1016/j.bbapap.2007.11.002

[42] Gajiwala KS, Feng J, Ferre R, Ryan K, Brodsky O, Weinrich S, et al Insights into the aberrant activity of mutant EGFR kinase domain and drug recognition. Structure. 2013;21: 209–219. doi: 10.1016/j.str.2012.11.014 23273428 10.1016/j.str.2012.11.014

[43] Gora A, Artur G, Jan B, Jiri D. Gates of Enzymes. Chem Rev. 2013;113: 5871–5923. doi: 10.1021/cr300384w 23617803 10.1021/cr300384w PMC3744840

[44] Gunasekaran K, Ma B, Nussinov R. Is allostery an intrinsic property of all dynamic proteins? Proteins. 2004;57: 433–443. doi: 10.1002/prot.20232 15382234 10.1002/prot.20232

[45] Pravda L, Lukáš P, Karel B, Vařeková RS, David S, Pavel B, et al Anatomy of enzyme channels. BMC Bioinformatics. 2014;15 doi: 10.1186/s12859-014-0379-x 25403510 10.1186/s12859-014-0379-x PMC4245731

[46] Chovancova E, Pavelka A, Benes P, Strnad O, Brezovsky J, Kozlikova B, et al CAVER 3.0: a tool for the analysis of transport pathways in dynamic protein structures. PLoS Comput Biol. 2012;8: e1002708 doi: 10.1371/journal.pcbi.1002708 23093919 10.1371/journal.pcbi.1002708 PMC3475669

[47] Zhou H-X, Wlodek ST, McCammon JA. Conformation gating as a mechanism for enzyme specificity. Proceedings of the National Academy of Sciences. 1998;95: 9280–9283. 10.1073/pnas.95.16.9280 PMC21329 9689071

[48] Ikura M, Ames JB. Genetic polymorphism and protein conformational plasticity in the calmodulin superfamily: two ways to promote multifunctionality. Proc Natl Acad Sci U S A. 2006;103: 1159–1164. doi: 10.1073/pnas.0508640103 16432210 10.1073/pnas.0508640103 PMC1360552

[49] Liu W, Ning J-F, Meng Q-W, Hu J, Zhao Y-B, Liu C, et al Navigating into the binding pockets of the HER family protein kinases: discovery of novel EGFR inhibitor as antitumor agent. Drug Des Devel Ther. 2015;9: 3837–3851. doi: 10.2147/DDDT.S85357 26229444 10.2147/DDDT.S85357 PMC4517520

[50] Monzon AM, Juritz E, Fornasari MS, Parisi G. CoDNaS: a database of conformational diversity in the native state of proteins. Bioinformatics. 2013;29: 2512–2514. doi: 10.1093/bioinformatics/btt405 23846747 10.1093/bioinformatics/btt405

[51] Berman HM, Westbrook J, Feng Z, Gilliland G, Bhat TN, Weissig H, et al The Protein Data Bank. Nucleic Acids Res. 2000;28: 235–242. 10592235 10.1093/nar/28.1.235 PMC102472

[52] Sali A, Blundell TL. Comparative protein modelling by satisfaction of spatial restraints. J Mol Biol. 1993;234: 779–815. doi: 10.1006/jmbi.1993.1626 8254673 10.1006/jmbi.1993.1626

[53] Forbes SA, Gurpreet T, Chai K, Mingming J, Sally B, Jennifer C, et al An Introduction to COSMIC, the Catalogue of Somatic Mutations in Cancer. NCI Nature Pathway Interaction Database. 2008; doi: 10.1038/pid.2008.3

[54] Le Guilloux V, Peter S, Pierre T. Fpocket: An open source platform for ligand pocket detection. BMC Bioinformatics. 2009;10: 168 doi: 10.1186/1471-2105-10-168 19486540 10.1186/1471-2105-10-168 PMC2700099

[55] Zhang X, Gureasko J, Shen K, Cole PA, Kuriyan J. Crystal Structure of the inactive EGFR kinase domain in complex with AMP-PNP [Internet]. 2006 doi: 10.2210/pdb2gs7/pdb

[56] Verkhivker G, Appelt K, Freer ST, Villafranca JE. Empirical free energy calculations of ligand-protein crystallographic complexes. I. Knowledge-based ligand-protein interaction potentials applied to the prediction of human immunodeficiency virus 1 protease binding affinity. Protein Eng. 1995;8: 677–691. 8577696 10.1093/protein/8.7.677

[57] Berrera M, Molinari H, Fogolari F. Amino acid empirical contact energy definitions for fold recognition in the space of contact maps. BMC Bioinformatics. 2003;4: 8 doi: 10.1186/1471-2105-4-8 12689348 10.1186/1471-2105-4-8 PMC153506

[58] Bickerton GR, Higueruelo AP, Blundell TL. Comprehensive, atomic-level characterization of structurally characterized protein-protein interactions: the PICCOLO database. BMC Bioinformatics. 2011;12: 313 doi: 10.1186/1471-2105-12-313 21801404 10.1186/1471-2105-12-313 PMC3161047

[59] Piovesan D, Minervini G, Tosatto SCE. The RING 2.0 web server for high quality residue interaction networks. Nucleic Acids Res. 2016;44: W367–74. doi: 10.1093/nar/gkw315 27198219 10.1093/nar/gkw315 PMC4987896

[60] Velec HFG, Gohlke H, Klebe G. DrugScore(CSD)-knowledge-based scoring function derived from small molecule crystal data with superior recognition rate of near-native ligand poses and better affinity prediction. J Med Chem. 2005;48: 6296–6303. doi: 10.1021/jm050436v 16190756 10.1021/jm050436v

[61] Lupyan D, Leo-Macias A, Ortiz AR. A new progressive-iterative algorithm for multiple structure alignment. Bioinformatics. 2005;21: 3255–3263. doi: 10.1093/bioinformatics/bti527 15941743 10.1093/bioinformatics/bti527

[62] Kuriyan J, Karplus M, Petsko GA. Estimation of uncertainties in X-ray refinement results by use of perturbed structures. Proteins. 1987;2: 1–12. doi: 10.1002/prot.340020102 3447165 10.1002/prot.340020102

[63] Baum BR. PHYLIP: Phylogeny Inference Package. Version 3.2 Joel Felsenstein. Q Rev Biol. 1989;64: 539–541.

[64] Margush T, McMorris FR. Consensusn-trees. Bull Math Biol. 1981;43: 239–244.

[65] Forbes SA, Beare D, Gunasekaran P, Leung K, Bindal N, Boutselakis H, et al COSMIC: exploring the world’s knowledge of somatic mutations in human cancer. Nucleic Acids Res. 2015;43: D805–11. doi: 10.1093/nar/gku1075 25355519 10.1093/nar/gku1075 PMC4383913

[66] Landrum MJ, Lee JM, Riley GR, Jang W, Rubinstein WS, Church DM, et al ClinVar: public archive of relationships among sequence variation and human phenotype. Nucleic Acids Res. 2014;42: D980–5. doi: 10.1093/nar/gkt1113 24234437 10.1093/nar/gkt1113 PMC3965032

[67] Kumar S, Nussinov R. Relationship between ion pair geometries and electrostatic strengths in proteins. Biophys J. 2002;83: 1595–1612. doi: 10.1016/S0006-3495(02)73929-5 12202384 10.1016/S0006-3495(02)73929-5 PMC1302257

[68] Kumar S, Nussinov R. Salt bridge stability in monomeric proteins. J Mol Biol. 1999;293: 1241–1255. doi: 10.1006/jmbi.1999.3218 10547298 10.1006/jmbi.1999.3218

[69] Barlow DJ, Thornton JM. Ion-pairs in proteins. J Mol Biol. 1983;168: 867–885. 6887253 10.1016/s0022-2836(83)80079-5

[70] Chovancova E, Pavelka A, Benes P, Strnad O, Brezovsky J, Kozlikova B, et al CAVER 3.0: a tool for the analysis of transport pathways in dynamic protein structures. PLoS Comput Biol. 2012;8: e1002708 doi: 10.1371/journal.pcbi.1002708 23093919 10.1371/journal.pcbi.1002708 PMC3475669

[71] Kingsley LJ, Lill MA. Substrate tunnels in enzymes: structure-function relationships and computational methodology. Proteins. 2015;83: 599–611. doi: 10.1002/prot.24772 25663659 10.1002/prot.24772 PMC4404149

[72] Huse M, Morgan H, John K. The Conformational Plasticity of Protein Kinases. Cell. 2002;109: 275–282. 12015977 10.1016/s0092-8674(02)00741-9

[73] Vijayan RSK, He P, Modi V, Duong-Ly KC, Ma H, Peterson JR, et al Conformational analysis of the DFG-out kinase motif and biochemical profiling of structurally validated type II inhibitors. J Med Chem. 2015;58: 466–479. doi: 10.1021/jm501603h 25478866 10.1021/jm501603h PMC4326797

[74] Gajiwala KS, Feng J, Ferre R, Ryan K, Brodsky O, Weinrich S, et al Insights into the aberrant activity of mutant EGFR kinase domain and drug recognition. Structure. 2013;21: 209–219. doi: 10.1016/j.str.2012.11.014 23273428 10.1016/j.str.2012.11.014

