# Correction: Pockets as structural descriptors of EGFR kinase conformations

**DOI:** 10.1371/journal.pone.0192815

**Published:** 2018-02-08

**Authors:** Marcia Anahi Hasenahuer, German Patricio Barletta, Sebastián Fernandez-Alberti, Gustavo Parisi, María Silvina Fornasari

In the Author Contributions section, María Silvina Fornasari should be listed as one of the persons involved in conceptualization.
