# Supplementary figures and images for: Pockets as structural descriptors of EGFR kinase conformations
Source: PLoS One. 2017 Dec 11;12(12):e0189147. doi: 10.1371/journal.pone.0189147 (PMC5724837; doi:10.1371/journal.pone.0189147)

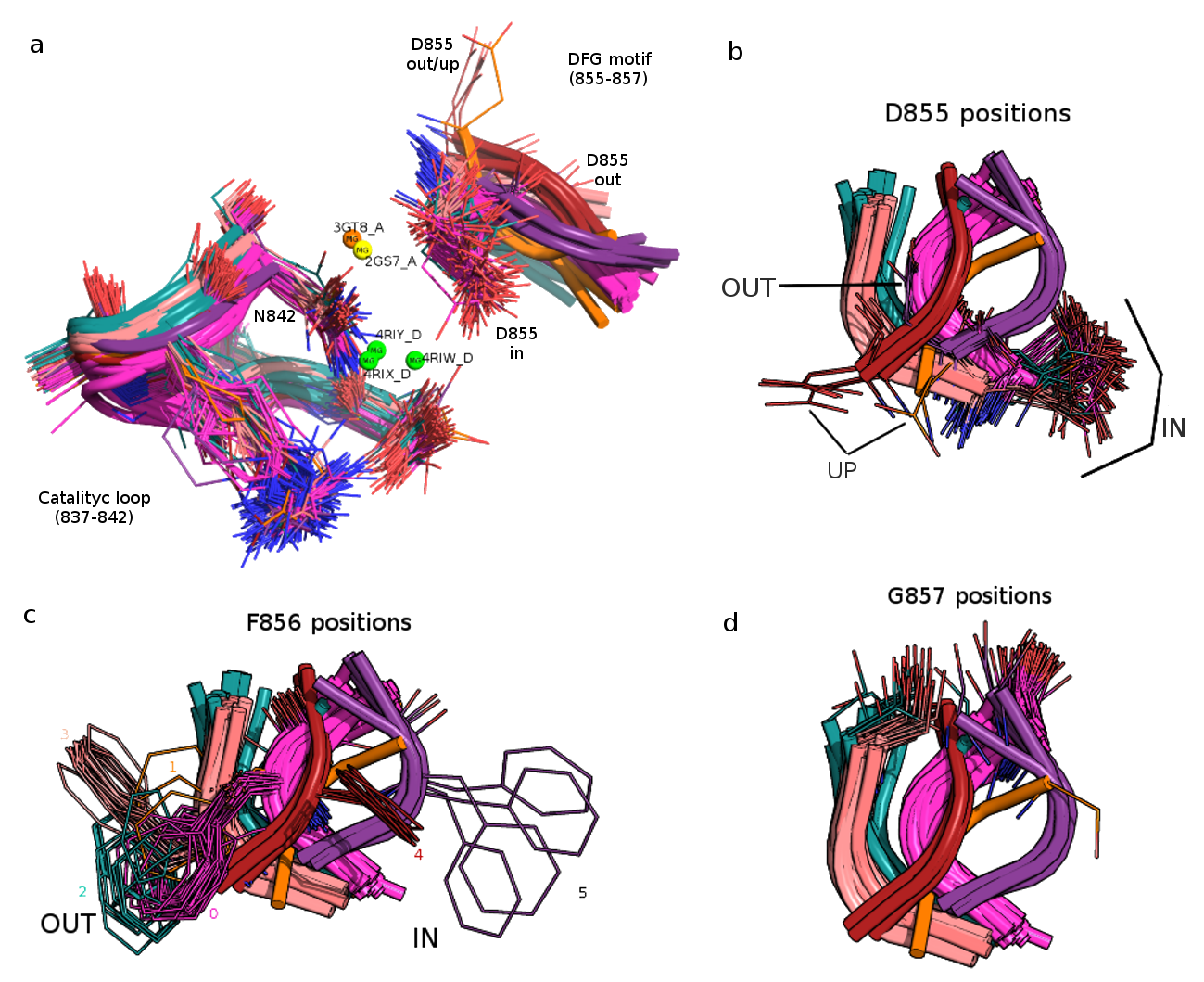

Supplement: S1 Fig — (A) D855 in—D855 out. (Dfg). Superposition of backbones of DFG motif and catalytic loop. Mg2+ ions correspond to the ones observed in inactive chains from symmetric dimers (PDB ids 3GT8 and 2GS7, as orange and yellow balls respectively) and active chains in asymmetric hetero-dimers (PDB ids 4RIX, 4RIY, 4RIW, as green balls). (B) Different D855 orientations (Dfg). IN, pointing to Mg2+ in active site. OUT and UP, two different orientations pointing away from the active site. (C) Alternative F856 positions (dFg). Numbers 0 to 5 represent the different categories used to divide conformations (a more detailed description is included in S1 Table). (D) G857 positions (dfG). (TIF) [file pone.0189147.s001.tif]

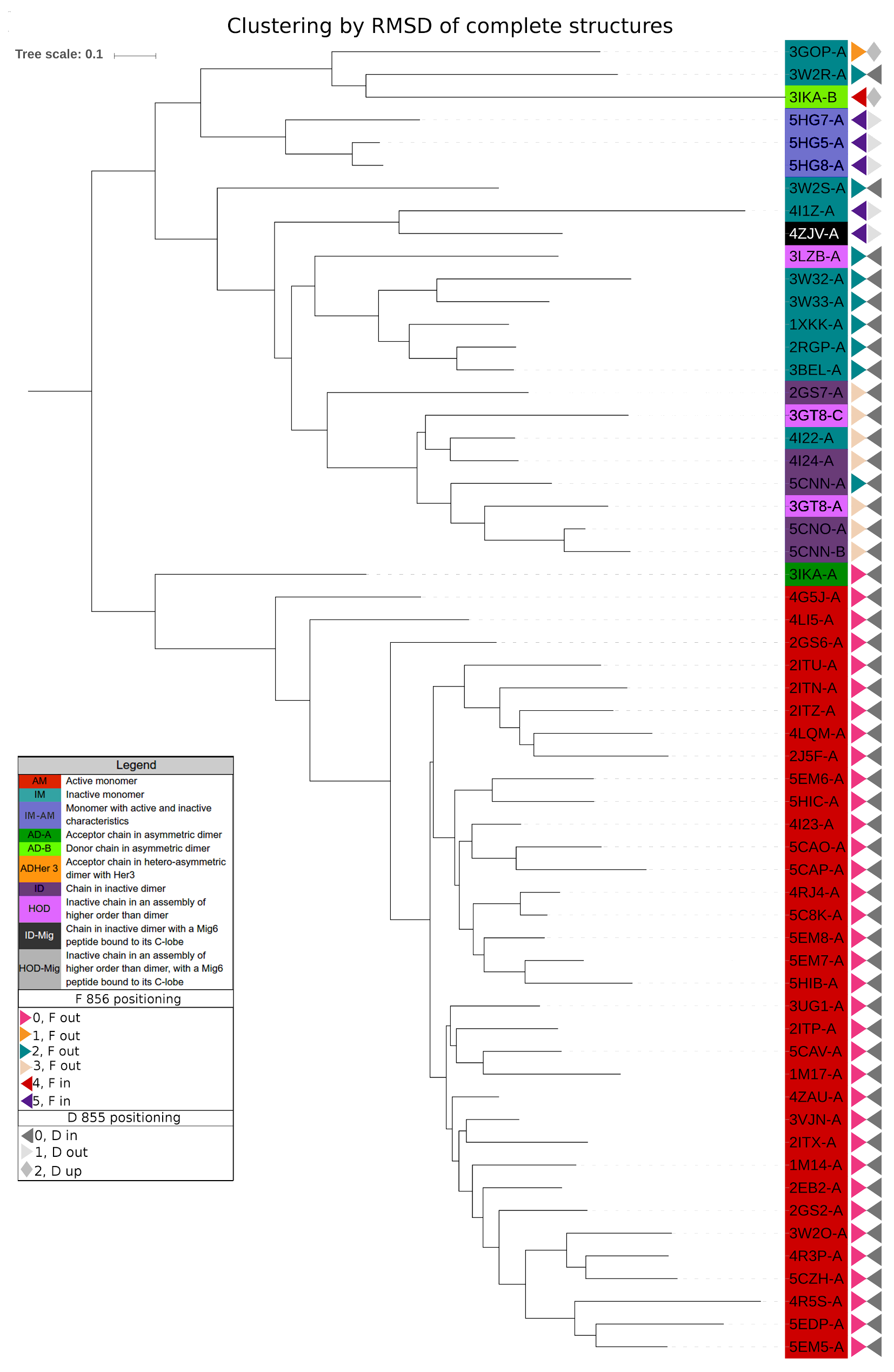

Supplement: S2 Fig — (TIF) [file pone.0189147.s002.tif]
